# Gut microbiota and the kidney-gut-skin axis in chronic kidney disease-associated pruritus: mechanisms and therapeutic implications

**DOI:** 10.3389/fcimb.2026.1811786

**Published:** 2026-05-20

**Authors:** Saiping Chen, Junyi Liu, Hongyi Ni, Fenggui Zhu, Hong Liu, Riyang Lin

**Affiliations:** 1 Department of Nephrology, Hangzhou Traditional Chinese Medicine Hospital of Zhejiang Chinese Medical University, Hangzhou, Zhejiang, China; 2 Key Laboratory of Kidney Disease Prevention and Control Technology, Hangzhou, Zhejiang, China

**Keywords:** chronic kidney disease-associated pruritus, gut-directed interventions, immune dysregulation, kidney-gut-skin axis, uremic toxins

## Abstract

Chronic kidney disease-associated pruritus (CKD-aP) is a highly prevalent and debilitating symptom in patients with chronic kidney disease (CKD) and end-stage kidney disease (ESKD), severely impairing quality of life, sleep quality, mental health, and clinical outcomes. Its pathogenesis is multifactorial and remains incompletely understood, involving chronic inflammation, immune imbalance, abnormal neuro-opioid pathways, mineral metabolism disorders and skin barrier damage. The kidney–gut–skin axis has attracted increasing attention as a novel theoretical framework to elucidate the roles of gut microbiota dysbiosis, gut-derived uremic toxins, intestinal barrier impairment and systemic inflammation in the development of CKD-aP. This review summarizes the traditional pathogenic mechanisms of CKD-aP, reviews recent advances linking gut microbial alterations to pruritus-related pathways, and systematically evaluates gut-targeted and metabolism-targeted interventions, including probiotics, prebiotics, synbiotics, AST-120, fecal microbiota transplantation, phytochemicals, Uremia Clearance Granules, and vitamin D-related strategies. Current evidence is mostly associative and is mainly derived from general CKD/ESKD populations, animal models, and *in vitro* studies; specific clinical validation in CKD-aP cohorts remains limited. Accordingly, gut microbiome-related mechanisms and interventions remain hypothetical and adjunctive, without established causal relationships or validated standard therapies for CKD-aP. Future studies are required to identify CKD-aP-specific pathological alterations, adopt longitudinal design and multi-omics analysis, conduct mechanistic verification, and perform randomized controlled trials with pruritus as a predefined primary endpoint.

## 1 Introduction

CKD-aP, historically referred to as uremic pruritus, is a common and distressing symptom in patients with CKD and ESKD. The prevalence of CKD-aP in patients with ESKD has been widely reported to vary substantially, ranging from 18% to 80% (Kim and Pollock, 2021). Such considerable heterogeneity may stem from differences in study populations, dialysis modalities, diagnostic standards, and pruritus evaluation approaches across relevant literature. These variations complicate direct comparisons between studies and highlight the need for standardized assessments in future research on CKD-aP. In addition, a multinational cross-sectional study of patients with CKD stages 3–5 demonstrated that up to 24% of participants reported moderate-to-severe pruritus (Sukul et al., 2019). This symptom exerts a significant adverse impact on patients’ physical and mental health as well as their quality of life. International studies have demonstrated that 60% of patients with moderate to severe CKD-aP suffer from sleep insufficiency, and the risk of fatigue in pruritic patients is 2 to 5.8 times higher than that in non-pruritic patients (Kimata et al., 2014; Agarwal et al., 2021; Sukul et al., 2021). Moreover, severe CKD-aP has been associated with poor clinical outcomes, which can elevate patients’ risk of depression and increase their all-cause mortality risk by 17% to 22% (Kim and Pollock, 2021). Given that the pathophysiological mechanisms underlying CKD-aP have not been fully elucidated, this symptom is frequently underrecognized and undertreated in clinical practice (Scherer et al., 2017). In recent years, research perspectives have shifted from the traditional notion that non-specific accumulation of uremic toxins induces pruritus to the exploration of multiple pathological mechanisms.

This narrative review was based on a structured search of PubMed, Web of Science, Embase, Scopus, CNKI, and Wanfang from database inception to May 2, 2026. Search terms included combinations of “CKD-aP”, “uremic pruritus”, “chronic kidney disease”, “end-stage kidney disease”, “gut microbiota”, “gut microbiome”, “kidney-gut-skin axis”, “uremic toxins”, “indoxyl sulfate”, “p-cresyl sulfate”, “short-chain fatty acids”, “lipopolysaccharide”, “IL-31”, “skin barrier”, “opioid receptor”, “vitamin D”, “iNOS”, “fecal microbiota transplantation”, “probiotics”, “prebiotics”, “synbiotics”, “AST-120”, “phytochemicals”, and “Uremia Clearance Granules”. Eligible studies included clinical, observational, interventional, review, meta-analytic, and mechanistic studies relevant to CKD-aP, CKD/ESKD-related microbiome alterations, gut-derived toxins, inflammatory pathways, skin barrier dysfunction, neural/opioid pathways, or gut-directed interventions. Because this is a narrative review, no PRISMA-based risk-of-bias assessment or quantitative meta-analysis was performed. Evidence was interpreted according to whether it came from CKD-aP patients, general CKD/ESKD cohorts, animal models, *in vitro* studies, or broader mechanistic literature.

## 2 Traditional and clinically recognized mechanisms of CKD-aP

### 2.1 Chronic inflammation and immune dysregulation

Systemic low-grade inflammation is frequently observed in CKD and ESKD and may provide an important background for CKD-aP. Progressive renal dysfunction is commonly accompanied by uremic toxin accumulation, mineral metabolism disorders, oxidative stress, and chronic inflammatory activation (Tinti et al., 2021; Zoccali and Mallamaci, 2023). These CKD-related alterations may contribute to innate and adaptive immune activation, but they should be interpreted primarily as features of the general CKD/ESKD inflammatory milieu rather than CKD-aP-specific mechanisms. In general CKD populations, expansion of proinflammatory monocyte subsets has been reported (Cormican et al., 2023). By contrast, more direct CKD-aP-related clinical evidence comes from studies showing that patients with CKD-aP have elevated serum levels of interleukin-2 (IL-2) and interleukin-6 (IL-6) compared with non-pruritic CKD or hemodialysis controls (Kimmel et al., 2006; Fallahzadeh et al., 2011). Other inflammatory mediators, including tumor necrosis factor-α(TNF-α) and interleukin-1β(IL-1β), have also been implicated in CKD-related inflammatory responses (Kim et al., 2019; Verduzco and Shirazian, 2020), but their direct contribution to CKD-aP severity remains incompletely defined. At the skin level, inflammatory mediators may interact with keratinocytes, immune cells, and cutaneous sensory nerve endings. Calcium phosphate (CaP) has been reported to activate itch-related signaling in an IL-6-dependent manner through upregulation of phosphorylated extracellular signal-regulated kinase (p-ERK) in experimental settings (Keshari et al., 2019). TNF-α and IL-6 may also participate in neuroimmune sensitization pathways; however, evidence directly linking these cytokines to Janus kinase 2/signal transducer and activator of transcription 3 (JAK2/STAT3)-mediated cutaneous itch signaling in CKD-aP remains indirect (Fallahzadeh et al., 2011). Therefore, this pathway should be interpreted as a biologically plausible mechanism rather than an established CKD-aP-specific cascade.

Mast cell activation has been observed in the skin of patients with CKD (Uppal et al., 2022). Although histamine is not considered the major mediator of CKD-aP, mast-cell-derived tryptase may activate protease-activated receptor 2 (PAR-2) in the skin and thereby participate in non-histaminergic itch signaling (Steinhoff et al., 1999).

Interleukin-31 (IL-31) represents one of the more directly relevant cytokines in CKD-aP. Elevated serum IL-31 levels have been reported in hemodialysis patients with CKD-aP, and IL-31 levels are associated with pruritus severity (Ko et al., 2014; Oweis et al., 2021; Świerczyńska et al., 2022). As a T helper 2 (Th2)-related pruritogenic cytokine, IL-31 can act on IL-31 receptor-expressing cutaneous sensory neurons and keratinocytes, potentially promoting nerve sensitization and the release of pruritogenic mediators such as nerve growth factor (NGF) (Datsi et al., 2021; Kabashima and Irie, 2021; Świerczyńska et al., 2022). Moreover, IL-31 expression has been reported to be more widely distributed throughout the epidermis in patients with CKD-aP rather than being restricted to the basal layer (Datsi et al., 2021). These findings suggest that CKD-aP should not be interpreted as a purely T helper 1 (Th1)-driven inflammatory condition. A more balanced model is that CKD-related systemic microinflammation may coexist with local cutaneous Th2/IL-31-related pruritogenic signaling.

Taken together, available evidence suggests that inflammation-related mechanisms in CKD-aP may involve systemic CKD-associated inflammation, dermal and epidermal immune responses, and cutaneous sensory nerve sensitization. However, the relative contribution of each pathway to CKD-aP onset and severity remains incompletely established, and indirect mechanisms should be interpreted cautiously.

### 2.2 Peripheral neuropathy and opioid pathway abnormalities

Peripheral neuropathy is a common complication in patients with CKD, mainly characterized by sensory disturbances (Arnold et al., 2022). Neuropathy in CKD-aP should be understood primarily as abnormalities in the peripheral pruritic afferent pathway, mainly localized to intraepidermal sensory nerve endings and peripheral afferent nerve fibers in the skin. With progressive deterioration of renal function, uremic toxin accumulation, oxidative stress, and chronic low-grade inflammation may coexist and contribute to peripheral sensory dysfunction (Schricker and Kimmel, 2021). These CKD-related disturbances may affect cutaneous sensory nerve function, lower activation thresholds, and promote peripheral sensitization (Verduzco and Shirazian, 2020). Consistently, abnormal expression of ion channels has been observed in the peripheral nerve endings of the skin in patients with CKD-aP, which may increase itch-related generator potentials and contribute to pruritus perception (Momose et al., 2019).

Dysbalance of peripheral opioid receptors constitutes another pathogenic mechanism underlying CKD-aP. Opioid receptors are not exclusively distributed in the central nervous system, but also participate in the regulation of the skin and local immune microenvironment (Bigliardi et al., 2009). When inflammation persists chronically, the pruritic-antipruritic balance of the opioid system is more readily disrupted (Wala-Zielińska et al., 2023). Patients with chronic kidney disease commonly exhibit persistent low-grade inflammation, and the associated inflammatory cytokines may contribute to pruritic signaling and may also modulate opioid pathways in peripheral tissues and sensory nerve endings, further disturbing local opioid homeostasis (Wala-Zielińska et al., 2023).

Available evidence indicates that opioid system imbalance in CKD-aP is predominantly localized to the cutaneous/peripheral opioid system. The most direct abnormality is downregulated expression of the κ-opioid receptor (KOR) in the skin. Studies have shown that skin KOR expression is significantly lower in hemodialysis patients with pruritus than in those without pruritus (Wieczorek et al., 2020). In contrast, no significant difference in skin μ-opioid receptor (MOR) expression has been observed (Wieczorek et al., 2020). KOR activation exerts an antipruritic effect (Cowan et al., 2015), whereas activation of the mu opioid receptor (MOR) can induce pruritus (Uppal et al., 2022). These findings suggest that impaired peripheral KOR-mediated antipruritic signaling may represent one component of CKD-aP pathophysiology (Cowan et al., 2015). Consistently, the ratio of β-endorphin to dynorphin A was also markedly decreased in CKD-aP patients (Wala-Zielińska et al., 2023), further supporting a shift toward impaired antipruritic regulation.

### 2.3 Skin barrier dysfunction and mineral deposition

Impaired skin barrier function is considered an important contributing factor to CKD-aP, and is commonly characterized by xerosis, altered stratum corneum structure, increased transepidermal water loss (TEWL), and, in some patients, mineral deposition. In CKD, impaired renal sodium and water regulation, reduced sweat and sebaceous gland activity, and changes in skin hydration may compromise epidermal barrier integrity (Waziri et al., 2019). Clinical studies have shown that epidermal water content is significantly lower in pruritic CKD patients, particularly those receiving hemodialysis, than in non-pruritic patients (Morton et al., 1996). Although xerosis may also occur in CKD patients without pruritus, it may increase susceptibility to pruritus by weakening barrier function, exposing cutaneous nerve endings, and enhancing sensitivity to external stimuli (Hu et al., 2019).

Mineral metabolism disorders, including hyperphosphatemia and calcium-phosphate imbalance, may also contribute to pruritus-related skin changes in CKD. Basic calcium phosphate (BCP) crystals can deposit in the skin and subcutaneous tissues, and experimental evidence suggests that these crystals may activate local inflammatory responses, including macrophage infiltration and cytokine release (Nadra et al., 2005). In CKD-aP patients, histopathological examinations have revealed calcium salt deposition in the dermal layer (Ruderman et al., 2020). These findings suggest that mineral deposition and local inflammation may contribute to cutaneous pruritogenic signaling, although the extent to which these changes directly determine CKD-aP severity remains incompletely defined.

## 3 The kidney-gut-skin axis in CKD-aP: biological rationale and clinical associations

### 3.1 Conceptual framework of the kidney-gut-skin axis

In CKD, declining renal function is associated with retention of urea and other metabolic waste products, some of which may diffuse into the intestinal lumen (Hobby et al., 2019). Within the gut, urea can be hydrolyzed by urease-producing bacteria into ammonia and ammonium hydroxide, potentially increasing luminal pH and altering the intestinal microenvironment (Rysz et al., 2021). In parallel, CKD-related factors such as dietary restrictions, constipation, slowed intestinal transit, polypharmacy, and repeated antibiotic exposure may further shape gut microbial composition (Sabatino et al., 2015). These changes are commonly characterized by enrichment of urease-producing and proteolytic bacteria and reduction of SCFA-producing commensal bacteria in CKD or ESKD populations (Tang et al., 2023).

SCFAs are important for enterocyte energy supply, tight-junction protein expression, and mucosal immune tolerance. Reduced SCFA availability may contribute to impaired intestinal barrier integrity (Peng et al., 2024). At the same time, enrichment of urease-producing and proteolytic bacteria may increase the generation of ammonia, endotoxins, and gut-derived uremic toxin precursors (Vaziri et al., 2012; Sabatino et al., 2015). However, intestinal dysbiosis and barrier dysfunction in CKD should be regarded as interrelated processes rather than a clearly established one-way causal sequence. Whether gut microbial dysbiosis precedes barrier dysfunction or directly contributes to CKD-aP remains uncertain.

Upon impairment of the intestinal barrier, lipopolysaccharide (LPS), microbial fragments, and gut-derived metabolites may gain easier access to the systemic circulation, thereby potentially contributing to systemic inflammation and metabolic disturbances (Lau et al., 2015; Mosterd et al., 2021). Such metabolic abnormalities are not merely a passive outcome of diminished renal clearance, but rather a joint consequence of altered microbial metabolic patterns and increased intestinal permeability. Gut-derived uremic toxins, such as indoxyl sulfate (IS) and trimethylamine N-oxide (TMAO), have been implicated in CKD progression and systemic metabolic burden (Ruderman et al., 2020; Rysz et al., 2021). These circulating metabolites and inflammatory mediators may also influence skin barrier function and itch-related signaling pathways (Qiao et al., 2024). Nevertheless, direct evidence that these gut-derived factors induce or exacerbate CKD-aP in humans remains limited.

In summary, intestinal microbial dysbiosis and barrier dysfunction are associated with renal function decline and may contribute to metabolite imbalance and systemic inflammation. These processes may provide a biological background relevant to CKD-aP, but they should not be interpreted as established causal mechanisms. Recent experimental work has suggested that gut microbial metabolites can regulate N^6^-methyladenosine (m^6^A) RNA methylation in spinal dorsal horn neurons and thereby influence itch-related gene expression (Jin et al., 2025). Although this finding provides an intriguing mechanistic clue for gut–nervous system communication in itch, its relevance to CKD-aP remains exploratory and has not been directly validated in CKD-aP patients.

### 3.2 Gut microbiota characteristics in CKD and CKD-aP

Several clinical studies in CKD patients (not specifically selected for pruritus) have reported reduced gut microbial diversity compared with healthy individuals: the relative abundances of Proteobacteria and Actinomycetes are increased, while those of Firmicutes and Bacteroidetes are decreased (Hobby et al., 2019; Chen et al., 2023). Moreover, these structural alterations in the microbiota correlate with the severity of renal impairment (Wang et al., 2015).

Building on these findings, recent studies have attempted to identify gut microbial signatures potentially associated with pruritus in hemodialysis patients with CKD-aP, rather than merely characterizing microbial changes in general CKD patients (Ko et al., 2025). One case-control study compared the fecal microbiota between maintenance hemodialysis (MHD) patients with and without pruritus. The results showed no significant difference in α-diversity between the two groups but distinct separation in β-diversity, suggesting similar overall microbial richness but different community structures (Ko et al., 2025). Taxonomic analysis revealed that Dialister, Pasteurellales, and Pasteurellaceae were relatively enriched in patients with CKD-aP, and the relative abundance of Dialister was positively correlated with pruritus severity (Ko et al., 2025).

These findings suggest that alterations in the gut microbiota of CKD-aP patients may not merely represent non-specific manifestations of advanced renal dysfunction. However, current evidence is primarily correlational and insufficient to establish causality. Enrichment of specific taxa does not equate to direct “drive” of pruritus, and there is currently insufficient evidence to define any genus or species as a direct causative microbe for CKD-aP (Ko et al., 2025). Future functional validation experiments—such as fecal microbiota transplantation, germ-free or antibiotic depletion models, targeted strain colonization, and metabolite tracing—are warranted to explore potential causal relationships between microbial alterations and CKD-aP.

In addition, this study and other similar investigations have methodological limitations: small sample sizes, and incomplete control or insufficient discussion of confounding factors that may influence the gut microbiome, including dialysis modality, dietary habits, constipation status, phosphate binders, proton pump inhibitors, and diabetes mellitus (Ko et al., 2025). To clarify the study populations, sequencing methods, main microbial findings, and limitations of existing human studies, representative microbiome-related studies are summarized in **Table 1**.

**Table 1:** Key human microbiome-related studies relevant to CKD, ESKD, and CKD-aP.

Study	Population	Cohort & control	Sequencing	Key findings	Relevance to CKD-aP	Main limitations
Ko et al., 2025	CKD-aP/hemodialysis	Case-control: HD patients with pruritus vs. HD without pruritus	16S rRNA	α-diversity unchanged; β-diversity distinct; *Dialister*, Pasteurellales, Pasteurellaceae enriched; *Dialister* correlates with itch severity	Most direct and relevant microbiome study for CKD-aP	Cross-sectional; only correlation, no causality; HD population only; confounders (diet, constipation, diabetes, phosphate binders, PPIs, antibiotics) not controlled
Ren et al., 2020	General CKD (not CKD-aP)	CKD n=159 vs. Healthy n=273	16S rRNA (OTU)	Diversity decreased; *Klebsiella*/Enterobacteriaceae ↑; *Blautia*/*Roseburia* ↓	Background: CKD causes dysbiosis and uremic toxin–related functions	No pruritus assessment; not CKD-aP specific; controls are healthy subjects
Chen et al., 2023	General CKD (not CKD-aP)	CKD subgroups vs. Healthy	16S rRNA + metabolomics	Dysbiosis and metabolite profiles differ by CKD etiology	Background: CKD microbiome is highly heterogeneous	Not CKD-aP specific; diabetes/hypertension may confound findings
Rossi et al., 2016	Predialysis CKD (not CKD-aP)	RCT, n=37	16S rRNA	Synbiotics ↑ *Bifidobacterium*; ↓ Ruminococcaceae; ↓ pCS	Proof of concept: gut intervention reduces uremic toxins	Pruritus not an endpoint; predialysis population
Wang et al., 2025	HD with sleep disturbance (not CKD-aP)	RCT, n=80	16S rRNA	Probiotics ↑ *Bifidobacterium*; ↓ TG5 and IS	Intervention changes microbiota and IS	Pruritus score unchanged; sleep as primary entry criterion
Wang et al., 2015	Peritoneal dialysis (not CKD-aP)	RCT, n=39	No microbiome sequencing	Probiotics ↓ TNF-α, IL-6, endotoxin	Indirect evidence: gut modulation reduces inflammation	PD population; pruritus not measured
Haghighat et al., 2020	HD (not CKD-aP)	RCT, n=75	No microbiome sequencing	Synbiotics ↓ inflammatory markers	Supports gut-kidney inflammation link	Pruritus not an endpoint
Laiola et al., 2025	General CKD longitudinal (not CKD-aP)	CKD cohort vs. Healthy	Shotgun metagenomics	Defined “toxic microbiome” producing uremic toxin precursors	Mechanistic background for gut-derived toxins	Pruritus not an outcome; not CKD-aP specific

CKD, chronic kidney disease; CKD-aP, chronic kidney disease-associated pruritus; ESKD, end-stage kidney disease; HD, hemodialysis; PD, peritoneal dialysis; RCT, randomized controlled trial; OTU, operational taxonomic unit.

This table summarizes representative human microbiome-related studies relevant to CKD, ESKD, and CKD-aP, rather than an exhaustive list of all available studies. Studies were classified according to their primary population and endpoint. CKD-aP-specific evidence refers to studies enrolling patients with pruritus or comparing pruritic and non-pruritic CKD/ESKD patients, whereas general CKD/ESKD evidence refers to studies in which pruritus was not a predefined outcome. Findings from general CKD/ESKD or dialysis cohorts should be interpreted as background evidence and should not be considered CKD-aP-specific unless pruritus-related analyses were performed. Most available human microbiome data remain cross-sectional or associative; therefore, microbial taxa or functional signatures listed in this table should be regarded as hypothesis-generating rather than causal biomarkers of CKD-aP. ↑: increase/upregulation; ↓: decrease/downregulation.

## 4 Proposed mechanisms linking gut microbial alterations to CKD-aP

### 4.1 Gut-derived uremic toxins and itch-related signaling

In CKD, alterations in the gut microbial ecosystem, including enrichment of urease-producing and proteolytic bacteria, have been described mainly in general CKD or ESKD populations rather than specifically in CKD-aP cohorts (Hobby et al., 2019). Under this condition, incompletely digested proteins and amino acids may be metabolized in the intestinal lumen into precursors such as indole and p-cresol, which are subsequently converted by host metabolism into protein-bound uremic toxins (PBUTs), including IS and pCS (Graboski and Redinbo, 2020). Because these toxins bind tightly to albumin and cannot be efficiently removed by conventional hemodialysis, they progressively accumulate in patients with CKD (Li et al., 2019; Świerczyńska-Mróz et al., 2023). Clinical studies have reported associations between PBUTs and pruritus-related phenotypes: serum IS and pCS levels have been evaluated in hemodialysis patients with CKD-aP (Świerczyńska-Mróz et al., 2023), and increased total pCS levels have been associated with pruritus severity in CKD patients (Pavlovitch et al., 1984). However, these findings remain primarily associative and do not establish that IS or pCS directly causes CKD-aP. Therefore, PBUTs should be regarded as plausible contributors to the pruritogenic milieu in CKD-aP rather than confirmed causal mediators.

One potential pathway involves the PAR-2- transient receptor potential vanilloid 1(TRPV1) axis in keratinocytes and cutaneous sensory signaling. Evidence from *in vitro* human keratinocyte experiments has shown that IS and pCS can increase PAR-2 mRNA and protein expression and promote NGF secretion under experimental conditions (Kim et al., 2021). In the broader itch literature, PAR-2 activation has been linked to non-histaminergic pruritic signaling through downstream pathways involving phospholipase C-inositol trisphosphate-Ca^2+^(PLC-IP3-Ca^2+^) signaling and TRPV1 activation in cutaneous sensory neurons (Davidson and Giesler, 2010; Bautista et al., 2013; Kempkes et al., 2014; Ubeysinghe et al., 2023). Therefore, it is biologically plausible that IS- and pCS-induced PAR-2 upregulation may enhance keratinocyte–nerve communication and contribute to peripheral itch sensitization in CKD-aP. However, this pathway should be interpreted cautiously, because most mechanistic evidence is derived from *in vitro* keratinocyte studies or general itch models rather than direct causal studies in CKD-aP patients. Notably, PAR-2 knockdown attenuated IS- and pCS-induced keratinocyte activation *in vitro*, and increased epidermal PAR-2 expression has been observed in skin samples from patients with CKD-aP compared with non-pruritic CKD patients (Kim et al., 2021). These findings support an association between uremic solutes, PAR-2 expression, and CKD-aP-related itch pathways, but they do not establish that IS or pCS directly causes CKD-aP *in vivo*.

A second plausible but indirect pathway involves IS-related inflammatory and immune signaling. IS has been identified as an endogenous agonist of the aryl hydrocarbon receptor (AhR) in experimental systems (Schroeder et al., 2010). *In vitro* studies using human macrophages suggest that IS may activate AhR-associated oxidative stress and downstream nuclear factor-κB (NF-κB) and mitogen-activated protein kinase (MAPK) signaling pathways signaling, thereby increasing the expression of pro-inflammatory cytokines such as TNF-α, IL-6, and IL-1β (Kim et al., 2017, 2019). IS has also been shown to activate AhR signaling and upregulate IL-6 transcription in human hepatocyte-based experimental models (Schroeder et al., 2010). These findings support a biologically plausible link between IS accumulation and systemic inflammatory activation in CKD or ESKD. From the perspective of CKD-aP, these cytokine-related pathways may contribute to a pro-pruritic inflammatory milieu by influencing keratinocyte activation, immune-cell recruitment, and peripheral sensory sensitization. For example, TNF-α-related signaling has been associated with CX3C chemokine ligand 1 (CX3CL1) expression and T-cell recruitment in experimental inflammatory settings (Kim et al., 2017). IL-6 modulates the balance between pro- and anti-inflammatory cytokines via the JAK2/STAT3 signaling pathway and is involved in neuropathic sensory sensitization. In a rat model of neuropathic pain, red nucleus-derived IL-6 activates the JAK2/STAT3 pathway, upregulates the expression of pro-inflammatory cytokines such as TNF-α and IL-1β in the spinal cord, and simultaneously downregulates anti-inflammatory factors including transforming growth factor-β (TGF-β) and interleukin-10 (IL-10), thereby mediating the initiation and maintenance of tactile allodynia (Yang et al., 2022). This mechanism offers a biologically plausible framework for understanding how inflammatory cytokines may contribute to peripheral itch sensitization in CKD-aP, but direct evidence for this pathway in CKD-aP remains lacking.

**Figure 1:**
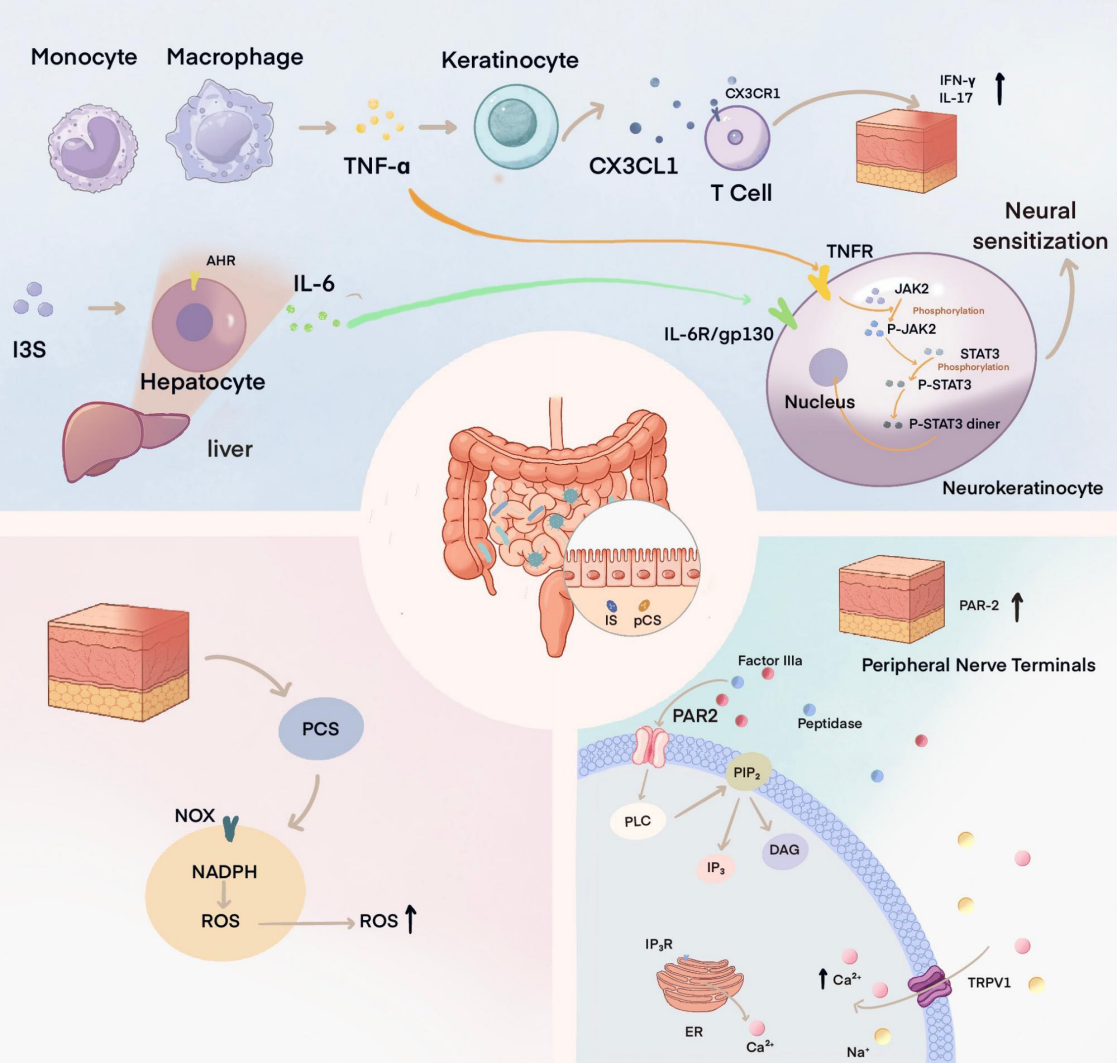
Proposed links between gut-derived uremic toxins and itch-related signaling in CKD-aP. This schematic summarizes potential pathways by which gut-derived protein-bound uremic toxins, particularly indoxyl sulfate (IS) and p-cresyl sulfate (pCS), may contribute to itch-related signaling in chronic kidney disease-associated pruritus (CKD-aP). Relatively supported observations include IS/pCS accumulation in CKD/ESKD, IS- and pCS-induced upregulation of protease-activated receptor 2 (PAR-2) in keratinocyte models, and increased epidermal PAR-2 expression in CKD-aP patients. Hypothetical or inferential links include direct IS/pCS-induced PAR-2–transient receptor potential vanilloid 1 (TRPV1) signaling in CKD-aP *in vivo*; IS-related aryl hydrocarbon receptor (AhR), nuclear factor-κB/mitogen-activated protein kinase (NF-κB/MAPK), tumor necrosis factor-α (TNF-α), interleukin-6 (IL-6), and JAK2/STAT3-mediated neural sensitization; CX3C chemokine ligand 1 (CX3CL1)/CX3C chemokine receptor 1 (CX3CR1)-mediated cutaneous immune recruitment; and pCS-related NADPH oxidase/reactive oxygen species (ROS)-driven skin barrier dysfunction. Thus, the image should be interpreted as a conceptual framework rather than a proven causal cascade; arrows indicate proposed biological associations or mechanistic directions rather than uniformly established causal relationships.

Another possible mechanism is related to pCS-associated oxidative stress. Experimental studies have shown that pCS can activate NADPH oxidase and increase reactive oxygen species (ROS) production in renal tubular epithelial cells and vascular endothelial cells (Watanabe et al., 2013, 2015). Although these findings are mainly derived from CKD-related cellular models rather than CKD-aP skin samples, they suggest a potential link between pCS accumulation and oxidative injury. In the skin, excessive ROS has been associated with keratinocyte dysfunction, impaired intercellular junctions, NF-κB/MAPK-related inflammation, and defective epidermal barrier repair (Choi et al., 2021). Therefore, pCS-related oxidative stress may plausibly contribute to a pro-pruritic cutaneous microenvironment by weakening barrier integrity and promoting peripheral sensory sensitization. However, direct evidence linking pCS-induced cutaneous ROS accumulation to CKD-aP severity remains limited (**Figure 1**).

### 4.2 Gut barrier dysfunction, LPS translocation, and systemic inflammation

In CKD and ESKD populations, gut microbiota dysbiosis, reduced SCFA production, and impaired intestinal barrier integrity have been associated with increased translocation of microbial-associated molecular patterns (MAMPs), such as LPS, from the intestinal lumen into the systemic circulation (Mosterd et al., 2021). This, together with the persistent elevation of gut-derived uremic toxins including IS and pCS, collectively establishes a pro-inflammatory systemic milieu.

LPS translocation may provide a biologically plausible connection between intestinal barrier dysfunction and systemic low-grade inflammation in CKD. After intestinal barrier impairment, LPS can interact with lipopolysaccharide-binding protein (LBP)/CD14 and the Toll-like receptor 4-myeloid differentiation factor 2 (TLR4-MD2) complex on monocytes, macrophages, and dendritic cells, leading to NF-κB/MAPK activation and production of inflammatory mediators such as TNF-α, IL-1β, IL-6, and interleukin-12 (IL-12) in experimental and general inflammatory contexts (Verzola et al., 2017; Tang et al., 2023). An IL-12-rich inflammatory milieu may favor Th1 differentiation through the STAT4/T-bet axis (Seder et al., 1993; Trinchieri, 1995; Szabo et al., 2003). However, direct evidence that gut-derived LPS drives Th1 polarization or itch severity in CKD-aP patients remains limited. Therefore, this pathway should be regarded as a plausible inflammatory framework rather than an established CKD-aP-specific mechanism.

In line with the concept of systemic low-grade inflammation in CKD, studies in general CKD populations have reported an expansion of proinflammatory monocyte subsets (Cormican et al., 2023), and increased proportions of Th1/Th17-related CD4^+^ T-cell phenotypes in peripheral blood (Basile et al., 2021; Zhang et al., 2024). However, these immune alterations have not been specifically validated as CKD-aP-specific features, and their direct relationship with gut barrier dysfunction, microbial translocation, or pruritus severity remains to be clarified.

This systemic inflammatory profile does not exclude the involvement of Th2-related pruritogenic pathways at the cutaneous level. In CKD-aP patients, elevated serum IL-31 levels have been reported, and IL-31 is widely recognized as an important pruritogenic cytokine (Świerczyńska et al., 2022). Because IL-31 can be produced by activated Th2 cells and is regulated by interleukin-4(IL-4)/signal transducer and activator of transcription 6 (STAT6)/GATA-binding protein 3 (GATA3) and NF-κB-related signaling in broader inflammatory and pruritic contexts (Bağci and Ruzicka, 2018; Takahashi et al., 2023), it may represent a link between systemic inflammation and local itch amplification. Therefore, CKD-aP may not be accurately characterized as a purely Th1- or Th2-driven condition. A more balanced model is that CKD-related systemic inflammation may coexist with local cutaneous Th2/IL-31-related pruritogenic signaling. In parallel, gut-derived metabolites such as SCFAs may influence mucosal immune regulation and Treg differentiation, as suggested by general immunological and microbiome studies (Tran et al., 2025). However, whether reduced SCFA-producing bacteria directly lead to Treg deficiency, Th2/Th17 skewing, or IL-31 upregulation in CKD-aP patients remains unproven. This connection should therefore be interpreted as a biologically plausible but speculative link between gut dysbiosis, immune imbalance, and pruritus.

### 4.3 Gut microbiota dysbiosis and metabolic disorders

The gut microbiota contributes to host metabolic homeostasis and immune regulation. In CKD and ESKD populations, gut microbial dysbiosis has been associated with altered microbial metabolic activity and a shift toward a more pro-oxidative and pro-inflammatory metabolic profile (Mafra et al., 2023). For example, reduced production of potentially protective metabolites, including SCFAs and tryptophan-derived indolepropionic acid, has been reported in CKD-related studies (Mafra et al., 2023), whereas several uremic or pro-oxidative metabolites, such as IS, advanced glycation end products (AGEs), and TMAO, are elevated in CKD or ESKD populations (Stubbs et al., 2016; Quadros et al., 2023; Zhang et al., 2025). These metabolic alterations may contribute to oxidative stress and impaired antioxidant defense in CKD. However, most of the available evidence comes from general CKD/ESKD cohorts or mechanistic studies rather than CKD-aP-specific populations. Therefore, the connection between these metabolic changes and pruritus perception should be interpreted cautiously. They may represent a systemic metabolic background that increases susceptibility to skin barrier dysfunction or peripheral sensory sensitization, but they should not be regarded as established CKD-aP-specific mechanisms.

From the perspective of the skin barrier, reduced SCFA production associated with CKD-related gut dysbiosis may represent a potential link between intestinal microbial alterations and cutaneous vulnerability. Experimental studies have shown that gut-derived SCFAs, particularly butyrate, can support epidermal barrier integrity by promoting keratinocyte metabolism and differentiation, increasing structural proteins such as filaggrin and loricrin, and regulating lipid components such as ceramides (Trompette et al., 2022). However, these findings are mainly derived from experimental or non-CKD-aP settings. Direct evidence demonstrating that reduced SCFAs cause skin barrier dysfunction or pruritus in CKD-aP patients remains limited. Therefore, SCFA deficiency should be interpreted as a biologically plausible contributor to skin barrier susceptibility rather than an established CKD-aP-specific mechanism.

**Figure 2:**
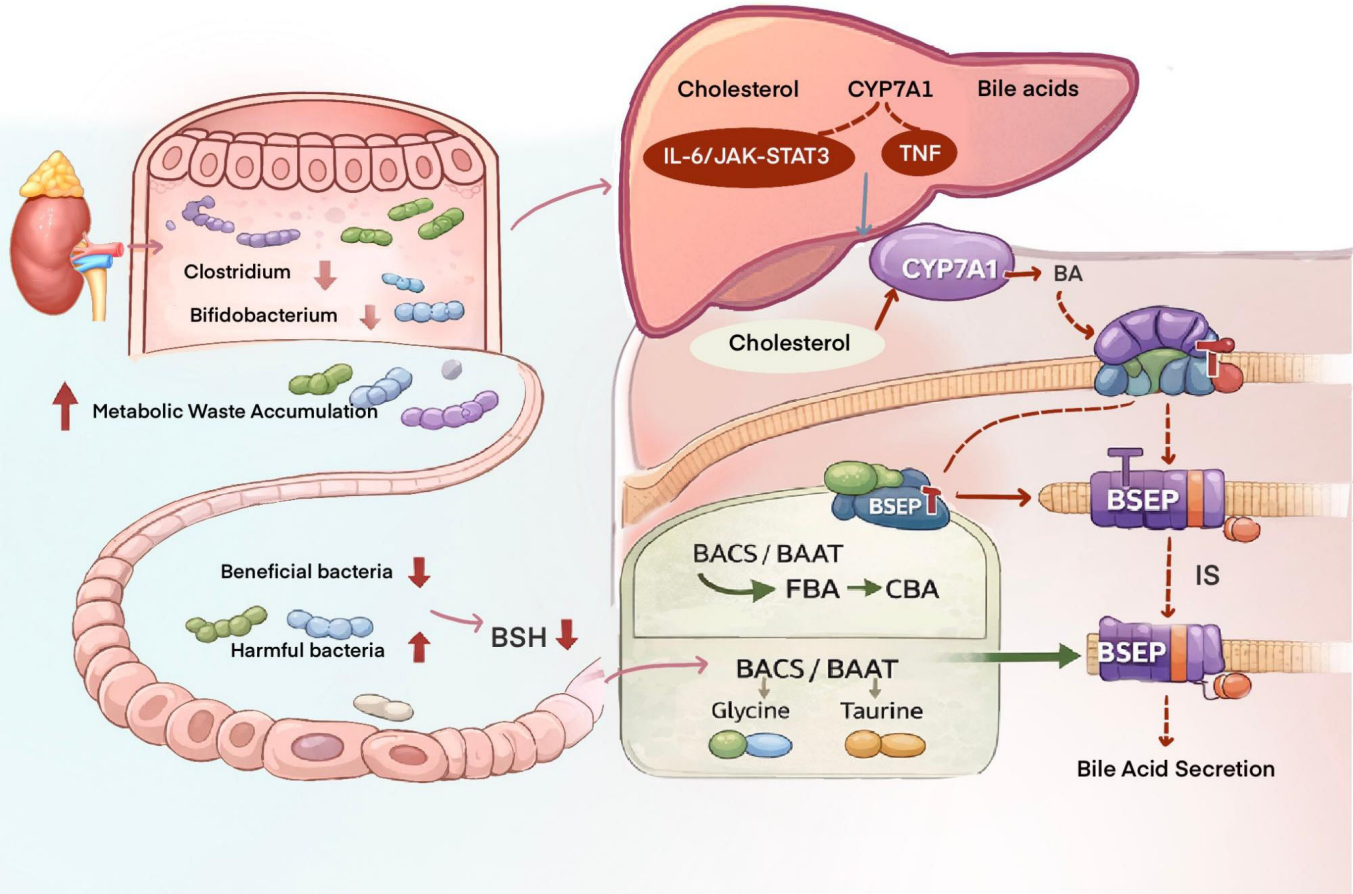
Proposed interactions among gut microbiota, bile acid metabolism, and vitamin D handling in CKD. This schematic illustrates potential interactions among renal function decline, gut microbial alterations, bile acid metabolism, hepatobiliary transport, vitamin D handling, and itch susceptibility in chronic kidney disease-associated pruritus (CKD-aP). Relatively supported observations include the accumulation of metabolic waste products in CKD, CKD-associated alterations in gut microbial composition, and the involvement of microbial bile salt hydrolase (BSH) activity in the conversion of conjugated bile acids (CBAs) to free bile acids (FBAs). In hepatic bile acid metabolism, bile acid-CoA synthetase (BACS) and bile acid-CoA:amino acid N-acyltransferase (BAAT) participate in the conjugation of free bile acids with glycine or taurine. Inflammatory cytokines, including interleukin-6 (IL-6) and tumor necrosis factor-α (TNF-α), may influence cholesterol 7α-hydroxylase (CYP7A1)-related bile acid synthesis in experimental or inflammatory settings. Indoxyl sulfate (IS) has also been proposed to affect hepatobiliary transport through interaction with the bile salt export pump (BSEP). Hypothetical or inferential links include the sequential pathway whereby CKD-related gut dysbiosis reduces BSH activity, disrupts bile acid transformation, impairs vitamin D absorption or metabolism, and thereby contributes to skin inflammation or itch susceptibility in CKD-aP. This sequence has not been directly established in CKD-aP patients. Therefore, the image should be interpreted as a conceptual framework rather than a proven causal cascade; arrows indicate proposed biological associations or mechanistic directions rather than uniformly established causal relationships.

**Figure 3:**
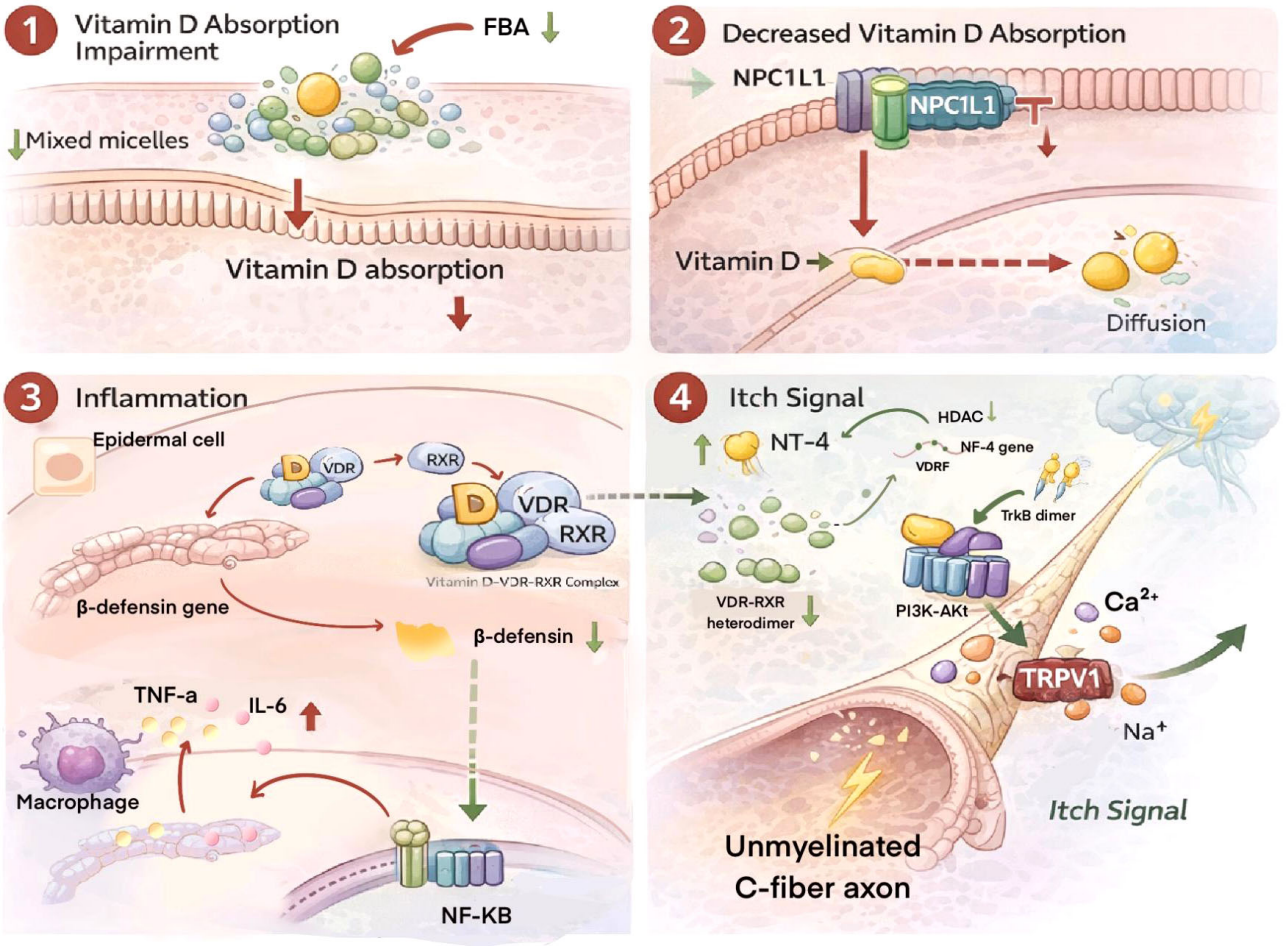
Proposed links between vitamin D-related pathways, skin inflammation, and itch susceptibility in CKD-aP. This schematic illustrates a proposed framework linking impaired vitamin D handling, skin barrier dysfunction, inflammatory signaling, and itch-related neural sensitization in chronic kidney disease-associated pruritus (CKD-aP). Relatively supported observations include the role of free bile acids (FBAs) and mixed micelles in the intestinal solubilization and absorption of fat-soluble molecules such as vitamin D, the involvement of Niemann-Pick C1-like 1 (NPC1L1) in intestinal vitamin D uptake, and the biological role of VDR signaling in epidermal differentiation, antimicrobial peptide expression, and immune regulation. Vitamin D/VDR signaling may also influence β-defensin expression and skin barrier defense. In broader inflammatory and dermatological contexts, pro-inflammatory cytokines such as tumor necrosis factor-α (TNF-α) and interleukin-6 (IL-6) can activate nuclear factor-κB (NF-κB)-related inflammatory pathways. Hypothetical or inferential links include the sequential pathway whereby CKD-related bile acid disturbance or reduced vitamin D absorption directly leads to VDR dysfunction, reduced β-defensin production, enhanced macrophage-derived inflammation, neurotrophin-4 (NT-4) upregulation, tropomyosin receptor kinase B (TrkB)-phosphatidylinositol 3-kinase-protein kinase B (PI3K-Akt) activation, transient receptor potential vanilloid 1 (TRPV1) sensitization, and ultimately itch signal transmission in CKD-aP. These links remain mechanistically plausible but have not been directly validated as a complete causal cascade in CKD-aP patients. Therefore, this image should be interpreted as a hypothesis-generating conceptual model rather than evidence of an established vitamin D-mediated itch pathway. Arrows indicate proposed biological associations or mechanistic directions rather than uniformly proven causal relationships.

Vitamin D metabolism may also intersect with gut microbial and metabolic pathways, but the evidence should be interpreted cautiously. Studies in broader clinical or experimental contexts suggest that the gut microbiota may influence vitamin D metabolism, intestinal absorption, and bile acid-related pathways (Tangestani et al., 2021; Ullah, 2025). In CKD patients, disturbances in vitamin D metabolism are common and may be further influenced by inflammation, altered bile acid handling, and uremic toxin accumulation. For example, IS-related effects on hepatobiliary transport and inflammatory cytokine–mediated regulation of vitamin D metabolic enzymes have been proposed as potential mechanisms (Li et al., 2024)(**Figure 2**). Nevertheless, the causal sequence linking gut dysbiosis, vitamin D deficiency, skin barrier impairment, and CKD-aP has not been directly established. Vitamin D deficiency has been associated with impaired epidermal differentiation, altered antimicrobial peptide expression, immune dysregulation, neural sensitization, and oxidative stress in experimental or non-CKD-aP studies (Pavlovitch et al., 1984; Tague et al., 2011; Oh, 2013; Patrick and Ames, 2015; Bishop et al., 2021; Karampinis et al., 2023; Zhu et al., 2024). In CKD-aP, small clinical studies and phototherapy-related observations suggest that vitamin D-related pathways may be relevant to pruritus (Jung et al., 2015; Gopinath et al., 2023), whereas evidence from atopic dermatitis or chronic urticaria should be considered supportive background rather than direct CKD-aP evidence (Goetz, 2011). Thus, vitamin D should be discussed as a potential modifier of skin barrier and immune function in CKD-aP, not as a confirmed mediator linking gut dysbiosis to pruritus (**Figure 3**).

Additional evidence from non-CKD-aP experimental models suggests that inducible nitric oxide synthase(iNOS)-related nitric oxide signaling may contribute to pruritic responses. For instance, increased iNOS expression has been reported in mice with cholestatic pruritus, and nitric oxide synthase inhibitors alleviated scratching behavior in this setting (Kim et al., 2023). However, such findings are limited to cholestatic pruritus and cannot be directly extrapolated to CKD-aP, and thus should be viewed only as mechanistic reference. Pro-inflammatory cytokines including IL-1β and TNF-α can upregulate iNOS expression in experimental systems (Marcus et al., 2003), but these observations are not specific to CKD-aP. In patients with CKD-aP, narrow-band ultraviolet B (NB-UVB) phototherapy has been shown to relieve pruritus, an effect associated with changes in serum vitamin D levels (Yamaoka et al., 2000; Kee et al., 2024). Experimental data also indicate that 25(OH)D may suppress iNOS expression (Garcion et al., 2003). Together, these findings imply potential crosstalk among inflammation, vitamin D-related pathways, and nitric oxide signaling in pruritus regulation, yet direct evidence for a functional iNOS-mediated pathway in CKD-aP remains scarce.

Overall, declining renal function is accompanied by gut dysbiosis, impaired intestinal barrier function, uremic toxin accumulation, systemic inflammation, and metabolic disturbances. These factors may collectively create a systemic and cutaneous environment that increases susceptibility to pruritus. However, the specific contributions of gut-derived toxins, LPS, vitamin D metabolism, and iNOS signaling to CKD-aP remain unclear. These pathways should therefore be considered biologically plausible and hypothesis-generating, rather than established causal mechanisms. To clarify the evidence hierarchy of these proposed pathways, **Table 2** summarizes the major mechanisms, evidence sources, and interpretation of their relevance to CKD-aP.

**Table 2:** Evidence map of proposed mechanisms linking gut microbiota to CKD-aP.

Proposed mechanism	Evidence source	Interpretation
PBUTs/IS/pCS (Schroeder et al., 2010; Watanabe et al., 2015; Kim et al., 2017; Graboski and Redinbo, 2020; Kim et al., 2021)	CKD/ESKD population cohorts, CKD-aP correlation studies, *in vitro* cell experiments	May contribute to the pro-pruritic microenvironment in CKD-aP, but the direct causal relationship for pruritus induction has not been confirmed *in vivo*
PAR-2-TRPV1 non-histaminergic pruritus pathway (Davidson and Giesler, 2010; Bautista et al., 2013; Kempkes et al., 2014; Kim et al., 2021)	*In vitro* keratinocyte experiments, observation of PAR-2 expression in skin of CKD-aP patients, mechanistic studies in general pruritus field	Has sufficient biological mechanistic support and is one of the most promising pruritogenic pathways in CKD-aP, but direct *in vivo* causal validation is still lacking
LPS-TLR4-NF-κB/MAPK systemic inflammatory pathway (Seder et al., 1993; Trinchieri, 1995; Szabo et al., 2003; Verzola et al., 2017)	General CKD/inflammatory disease models, non-CKD-aP specific studies	Only serves as a common framework for systemic inflammation in CKD, with no evidence supporting it as a specific pathogenic mechanism of CKD-aP
Th1/Th2/IL-31 immune imbalance pathway (Ko et al., 2014; Oweis et al., 2021; Zhang et al., 2024)	Direct clinical evidence of IL-31 in CKD-aP patients, Th1/Treg-related studies extrapolated from non-CKD-aP settings	CKD-aP is not driven by Th1 or Th2 alone, but more likely results from the combined effect of systemic inflammation and local IL-31 pruritogenic pathway
Short-chain fatty acids (SCFAs)-skin barrier function axis (Trompette et al., 2022; Peng et al., 2024)	Experimental animal studies, non-CKD-aP skin barrier related studies	Biologically plausible, but direct evidence linking reduced SCFAs to impaired skin barrier and aggravated pruritus in CKD-aP is still limited
Vitamin D/iNOS related pruritus pathway (Garcion et al., 2003; Jung et al., 2015; Gopinath et al., 2023)	Small-sample clinical studies in CKD-aP, non-CKD-aP mechanistic studies	Only an exploratory association, the direct causal chain between vitamin D, gut microbiota and CKD-aP has not been confirmed
m^6^A gut-spinal itch axis (Jin et al., 2025)	Animal experiments, *in vitro* mechanistic studies	Only a hypothesis-generating mechanism, without any clinical or *in vivo* evidence supporting its role in CKD-aP

CKD, chronic kidney disease; CKD-aP, chronic kidney disease-associated pruritus; PBUTs, protein-bound uremic toxins; IS, indoxyl sulfate; pCS, p-cresyl sulfate; PAR- 2, protease−activated receptor 2; TRPV1, transient receptor potential vanilloid 1; LPS, lipopolysaccharide; TLR4, Toll−like receptor 4; NF-κB, nuclear factor κB; MAPK, mitogen−activated protein kinase; SCFAs, short−chain fatty acids; iNOS, inducible nitric oxide synthase; m^6^A, N^6^- methyladenosine.

This table provides an evidence map of proposed mechanisms linking gut microbiota-related alterations to CKD-aP. The mechanisms listed include both relatively supported observations and inferential or hypothesis-generating pathways. “Evidence source” indicates whether the supporting data are derived from CKD-aP patients, general CKD/ESKD populations, animal models, *in vitro* experiments, or broader mechanistic literature. Mechanisms supported by general CKD/ESKD studies, non-CKD-aP disease models, or *in vitro* experiments should not be interpreted as established CKD-aP-specific causal pathways. References shown in the table are representative rather than exhaustive. Overall, the table is intended to clarify evidence strength and biological plausibility, not to imply that gut microbial dysbiosis directly causes CKD-aP.

## 5 Gut-directed and metabolism-directed interventions for CKD-aP

Based on the observed associations among gut microbial alterations, uremic toxin burden, systemic inflammation, and CKD-aP, several gut-directed or metabolism-directed interventions have been explored. These include fecal microbiota transplantation (FMT), probiotics, synbiotics, prebiotics, dietary fiber, AST-120, Uremia Clearance Granules, phytochemicals, and vitamin D-related or hormone-like approaches. However, these interventions differ substantially in their mechanisms, evidence levels, and clinical relevance to CKD-aP. Some approaches, such as AST-120 and Uremia Clearance Granules, have human CKD-aP-related data with pruritus outcomes, whereas probiotics, synbiotics, prebiotics, and dietary fiber are mainly supported by CKD or dialysis studies in which pruritus was not usually a predefined primary endpoint. In contrast, FMT, phytochemicals, and many vitamin D-related or hormone-like approaches remain largely preclinical, indirect, or exploratory in relation to CKD-aP. Therefore, these strategies should be regarded as potential adjunctive approaches rather than established CKD-aP treatments, and should be clearly distinguished from conventional management and approved antipruritic therapies. As summarized in **Table 3**, their evidence levels vary according to study population, pruritus endpoint, and evidence source.

**Table 3:** Summary of evidence for gut microbiota-targeted interventions in CKD-aP.

Intervention	Representative evidence	Preclinical CKD models	Pruritus endpoint	Evidence level	Main findings	Key limitations/interpretation
FMT	Preclinical CKD models (Barba et al., 2020; Wang et al., 2020)	CKD animal models; fecal microbiota from healthy donors or ESKD patients	No	Animal/preclinical evidence; hypothetical CKD-aP relevance	FMT or microbiota transfer may influence gut barrier integrity, uremic toxin generation, and systemic inflammation in CKD models	No clinical trial has evaluated FMT for CKD-aP; donor selection, infection risk, safety in immunocompromised CKD/dialysis patients, long-term engraftment, and regulatory issues remain unresolved
Probiotics/synbiotics	CKD, PD, and HD studies (Wang et al., 2015; Haghighat et al., 2020; Huang et al., 2021; Wang et al., 2025)	PD patients, HD patients, and experimental CKD models	Mostly no; one HD study assessed pruritus but showed no significant improvement	Human CKD/dialysis studies without definitive CKD-aP efficacy; animal/preclinical evidence	May reduce endotoxin, inflammatory markers, and indoxyl sulfate, and may modify gut microbiota composition	Most studies were not designed for CKD-aP; pruritus was usually not the primary endpoint; strain, dose, and duration vary; safety should be monitored in immunocompromised CKD/dialysis patients
Prebiotics, oligosaccharides, and dietary fibers	CKD trials and preclinical studies (Rossi et al., 2015, 2016; Mocanu et al., 2021; Ranganathan and Anteyi, 2022; Beau et al., 2025)	Predialysis CKD patients; HD patients; *in vitro* SHIME model; uremic rodent models	NO	Human CKD trials without pruritus endpoint; animal/*in vitro* evidence	May shift intestinal fermentation from proteolytic toward saccharolytic metabolism, reduce IS/pCS, increase Bifidobacterium, and support SCFA-producing bacteria	No direct CKD-aP efficacy data; dietary fiber strategies require individualized monitoring of potassium, phosphorus, protein-energy status, gastrointestinal tolerance, constipation, diabetes, and medications
AST-120	Human HD study and CKD background evidence (Yamaguchi et al., 2017; Wu et al., 2024)	Hemodialysis patients; CKD populations	Yes; VAS pruritus score	Yes; VAS pruritus score	Four-week AST-120 treatment was associated with reduced pruritus VAS score, lower serum IS, and reduced TNF-α	Relatively more direct clinical relevance, but evidence remains limited by short duration, limited sample size, and lack of long-term validation; efficacy in non-dialysis CKD or broader CKD-aP populations remains unclear
UCG	Meta-analysis and preclinical studies (Ji et al., 2020; Lu et al., 2021)	Patients described as having uremic pruritus or CKD-aP; CKD animal/preclinical models	Yes; VAS and response rate in clinical studies	Human CKD-aP-related evidence, but with methodological concerns; preclinical mechanism support	Meta-analysis reported reduced pruritus VAS score and improved response rate; preclinical studies suggest possible effects on gut barrier, microbiota, inflammation, and uremic milieu	Trial quality, placebo control, blinding, formulation heterogeneity, small sample size, publication bias, reproducibility outside East Asia, and CKD safety monitoring require further evaluation
Phytochemicals	Preclinical and mechanistic studies (Mirmohammadali and Rosenkranz, 2023; Li et al., 2025)	*In vitro* fermentation systems; animal models; general CKD/gut–kidney-axis studies	NO	Preclinical/*in vitro* evidence; hypothesis-generating relevance	Polyphenols, flavonoids, and related compounds may modulate gut microbiota, support SCFA-producing bacteria, reduce oxidative stress, and protect epithelial barrier function	No CKD-aP-focused RCTs; effective dose, bioavailability, renal handling, herb–drug interactions, product quality, electrolyte effects, and nephrotoxic risks remain uncertain
Active vitamin D	Small CKD-aP study, phototherapy observation, and RCT evidence (Bikle, 2012; Shirazian et al., 2013; Jung et al., 2015; Kee et al., 2024)	Small CKD-aP study, phototherapy observation, and RCT evidence	Yes in some studies; evidence mixed	Limited human CKD-aP evidence; indirect mechanistic evidence	Limited human CKD-aP evidence; indirect mechanistic evidence	Not a core microbiome-based therapy; relationship with gut dysbiosis remains indirect; vitamin D analogues require monitoring of calcium, phosphate, PTH, 25(OH)D, and vascular calcification risk

CKD-aP, chronic kidney disease-associated pruritus; CKD, chronic kidney disease; ESKD, end-stage kidney disease; FMT, fecal microbiota transplantation; HD, hemodialysis; PD, peritoneal dialysis; VAS, visual analog scale; IS, indoxyl sulfate; pCS, p-cresyl sulfate; SCFA, short-chain fatty acid; UCG, Uremia Clearance Granules; PTH, parathyroid hormone; RCT, randomized controlled trial.

Evidence level was assigned according to the study population and endpoint. Human CKD-aP studies with pruritus outcomes were considered more direct evidence, whereas CKD/ESKD studies without pruritus endpoints, animal studies, *in vitro* studies, and mechanistic hypotheses were considered indirect or exploratory evidence. References listed in the table are representative rather than exhaustive.

### 5.1 Fecal microbiota transplantation

FMT involves transferring feces from healthy donors into the patient’s gastrointestinal tract to modify the gut microbial community. It has been proven effective for conditions such as recurrent Clostridium difficile colitis (Xu et al., 2015), and is also being explored for metabolic and autoimmune diseases. In the context of CKD, FMT is still in the experimental stage, but conceptually, it may influence microbial diversity and function in the gut, may affect uremic toxin generation and inflammatory signaling in CKD models and inflammatory signals (Bian et al., 2022; Laiola et al., 2025). Animal studies provide preliminary preclinical support: an experiment transplanted the fecal microbial community of healthy mice onto germ-free CKD mice, and observed accumulation of uremic toxins and attenuation of renal damage (Barba et al., 2020). Notably, the IS and pCS levels of germ-free CKD mice were significantly lower than those of conventional CKD mice, supporting the contribution of gut microbiota to the generation of these toxins (Barba et al., 2020). In a 2020 study, Wang et al. performed fecal microbiota transplantation of fresh fecal samples from ESKD patients and healthy human donors into CKD mice; mice that received fecal microbiota from healthy human donors exhibited improved intestinal barrier function and reduced systemic inflammation (Wang et al., 2020). These findings suggest that gut microbial communities can influence toxin generation, barrier integrity, and inflammatory status in CKD models. However, they do not demonstrate that FMT improves pruritus or modifies CKD-aP-specific mechanisms in humans. Thus, FMT should be described as an exploratory gut-directed strategy with potential mechanistic relevance to the kidney–gut axis, rather than an established or near-term treatment for CKD-aP. Major unresolved issues include donor selection, infection transmission risk, safety in immunocompromised CKD or dialysis patients, long-term engraftment, regulatory oversight, and the need for trials with pruritus-specific endpoints.

### 5.2 Probiotics and synbiotics

Probiotics and synbiotics have been investigated as gut-directed approaches to modulate microbiota composition, uremic toxin generation, and inflammatory status in CKD and dialysis populations. However, most available studies were not designed specifically for CKD-aP, and pruritus was usually not the primary endpoint. For instance, Lactobacillus paracasei and Lactobacillus plantarum have been identified as uremia-targeted kidney-protective probiotics (Huang et al., 2021). These probiotics can reverse intestinal dysbiosis and restore symbiotic SCFA-producing bacteria, thereby improving intestinal barrier integrity and reducing systemic inflammation (Huang et al., 2021). These findings provide preclinical support for microbiome modulation in CKD, but they do not directly demonstrate antipruritic efficacy in CKD-aP. In experimental models, administration of probiotics has been shown to lower serum indoxyl sulfate and downregulate pro-fibrotic and pro-inflammatory genes in renal tissue (Huang et al., 2021). Clinically, small-scale trials in CKD patients have indicated that probiotics can reduce levels of inflammatory cytokines and endotoxins. A study on peritoneal dialysis patients found that 6 months of probiotic treatment significantly reduced serum levels of TNF-α, IL-5, IL-6, and endotoxin (Wang et al., 2015). In a randomized trial conducted in hemodialysis patients, a 6-month treatment with synbiotics (probiotics + prebiotic fiber) was found to be more effective than using probiotics alone in reducing inflammatory markers and circulating endotoxins (Haghighat et al., 2020). A recent randomized cohort study showed that blood dialysis patients with sleep disorders who received 2 packs of freeze-dried Lacticaseibacillus rhamnosus Lcr35 for 12 weeks could significantly improve the enrichment of the intestinal microbiota, manifested by increased abundance of Bifidobacterium, Prevotella, and Fusobacterium, and reduced TG5 abundance; this reduction in TG5 levels was correlated with decreased serum IS levels. However, in this study, there was no significant change in the patients’ pruritus scores (Wang et al., 2025). Taken together, probiotics and synbiotics may influence inflammation, endotoxin burden, uremic toxin levels, and gut microbial composition in CKD or dialysis patients. Nevertheless, direct evidence supporting their efficacy for CKD-aP remains limited. Future trials should use predefined pruritus endpoints, standardized strains and doses, adequate follow-up, and careful safety monitoring, particularly in immunocompromised CKD or dialysis patients.

### 5.3 Prebiotics, oligosaccharides and dietary fibers

Prebiotics refer to substrates that can be selectively utilized by host beneficial microorganisms and confer health benefits to the host, such as chicory root fiber and low-molecular-weight galactooligosaccharides. These prebiotic substrates can selectively stimulate the growth of intestinal beneficial bacteria, such as Bifidobacterium (Gibson et al., 2017). In patients with CKD, increasing dietary fiber intake or administering prebiotic supplements can shift intestinal metabolism from proteolysis to glycolysis, thereby reducing the production of indoles and phenolic compounds (Rossi et al., 2015; Ranganathan and Anteyi, 2022). Clinical studies have confirmed that pre-dialysis CKD patients supplemented with prebiotics (e.g., resistant starch or inulin) exhibit significantly reduced serum levels of indoxyl sulfate and p-cresyl sulfate, altered intestinal microbiome composition, increased enrichment of Bifidobacterium, and reduced abundance of the Ruminococcaceae family (Rossi et al., 2016). However, these studies were conducted in CKD populations rather than CKD-aP-specific cohorts, and pruritus was not the primary endpoint. In the latest research, a CKD-specific polybiotic (SynCKD) was formulated by selecting a uremic toxin-nonproducing probiotic strain (Lactobacillus johnsonii NCC533) and combining it with prebiotics (1% fructooligosaccharides) and postbiotics (1% short- and medium-chain triglycerides (C4-C8), a butyrate source). Administration of SynCKD for 6–8 weeks in two rodent models of uremia resulted in reduced plasma uremic toxins levels and improved renal function; metagenomic analysis revealed a decrease in microbial genes involved in tryptophan/tyrosine degradation, suggesting an improvement in intestinal ecological balance (Beau et al., 2025). Currently, many nephrologists advocate a plant-based, low-protein, high-fiber diet for CKD management to better control nitrogen balance, acid-base metabolism, and bone mineral disorders, reduce uremic toxin production, and slow CKD progression (Mocanu et al., 2021). Increased fiber and phytochemical intake may also support SCFA-producing bacteria and improve gut microbial ecology (Gibson et al., 2017; Mocanu et al., 2021). However, direct clinical evidence linking dietary fiber or prebiotic interventions to pruritus relief in CKD-aP remains limited. Therefore, dietary fiber optimization may be considered a supportive and individualized strategy rather than an established antipruritic treatment. In CKD and dialysis patients, such interventions should be implemented with attention to potassium and phosphorus load, protein-energy status, gastrointestinal tolerance, constipation, diabetes, and concurrent medications.

### 5.4 Oral adsorbent (AST-120)

AST-120 (brand name Kremezin) is an oral activated carbon preparation that can bind to the precursors of uremic toxins in the intestines, preventing their absorption. It mainly targets indole in the colon, thereby reducing serum indoxyl sulphate (Yamaguchi et al., 2017). AST-120 has been used in Japan for many years to slow the progression of CKD, and recent studies have shown that it also improves uremic pruritus (Wu et al., 2024). In a 2024 trial by Wu et al., compared to the control group, 4-week AST-120 treatment in hemodialysis patients resulted in a significant reduction in the VAS score for pruritus. This improvement corresponded to a 50% decrease in serum IS levels and a 50% decrease in TNF-α in the treatment group (Wu et al., 2024). It is notable that the degree of IS reduction is related to the reduction in PTH, indicating broader metabolic benefits (Wu et al., 2024).These findings suggest that AST-120 may reduce pruritus severity in hemodialysis patients, possibly in association with reductions in IS and inflammatory markers. However, the evidence remains limited by short treatment duration, limited sample size, and lack of long-term validation. Therefore, AST-120 represents a relatively more clinically supported gut/metabolism-directed strategy, but it should not be interpreted as definitive proof that intestinal toxin reduction universally improves CKD-aP (Yamaguchi et al., 2017).

### 5.5 Traditional Chinese medicine: uremia clearance granules

Uremia Clearance Granules (UCG), a multi-herb traditional Chinese medicine formula containing components such as rhubarb (Rheum genus) and astragalus, have been explored as an adjunctive therapy for CKD-aP. A 2021 meta-analysis of clinical studies reported that UCG was associated with reduced pruritus severity, as measured by VAS score, and an increased overall response rate in patients described as having uremic pruritus or CKD-aP (Lu et al., 2021). Some included studies also reported reductions in BUN, serum creatinine, PTH, IL-6, and TNF-α (Lu et al., 2021). These findings suggest that UCG may influence the uremic and inflammatory milieu in CKD-aP. However, the strength of this evidence should be interpreted cautiously because many included trials had limitations related to study quality, placebo control, blinding, heterogeneity of herbal formulations, small sample size, and potential publication bias. Experimental and pharmacological studies provide possible mechanistic support for some UCG components, particularly rhubarb. In CKD animal models or preclinical settings, rhubarb-based interventions have been reported to modulate gut microbiota, increase Lactobacillus and SCFA-producing bacteria, upregulate intestinal tight-junction proteins such as ZO-1 and occludin, and reduce LPS-related inflammation (Ji et al., 2020; Zhang et al., 2023). These findings are mechanistically relevant to the gut–kidney axis, but they should not be interpreted as direct proof that UCG alleviates CKD-aP through microbiome modulation in humans. Similarly, studies showing changes in intestinal microbiota after sennoside A administration provide supportive pharmacological background rather than CKD-aP-specific clinical evidence (Cui et al., 2019). More rigorous placebo-controlled studies (with clear formulations) are needed, but UCG provides a novel multi-target approach for CKD-aP, combining microbiome science with traditional medicine.

Therefore, UCG may be considered a potentially useful adjunctive approach for CKD-aP, particularly in settings where it is already clinically used. Nevertheless, its efficacy, optimal formulation, active components, safety profile, and reproducibility outside East Asian populations require confirmation in rigorously designed, placebo-controlled, blinded, multicenter trials with standardized preparations and pruritus-specific endpoints. Safety monitoring is especially important in CKD patients, given the potential risks of electrolyte disturbance, gastrointestinal adverse effects, herb–drug interactions, and variability in herbal product quality.

### 5.6 Phytochemicals

Phytochemicals are non-nutritive secondary metabolites synthesized by plants. They exert anti-inflammatory, antioxidant, intestinal barrier-protective, and antipruritic effects, and have also been recognized as promising microecological regulatory strategies in recent years (Mirmohammadali and Rosenkranz, 2023). On the one hand, polyphenols, flavonoids, and other plant-derived active ingredients promote the proliferation of short-chain fatty acid-producing commensal bacteria and reduce proteolytic fermentation substrates and their harmful metabolites (Pan et al., 2023; Nemzer et al., 2025). On the other hand, these compounds reduce systemic dissemination of gut-derived toxins, endotoxins, and proinflammatory cytokines through antioxidant, anti-inflammatory, and epithelial barrier-protective effects (Zhang et al., 2022; Li et al., 2025). In other words, phytochemicals are not direct antipruritic agents targeting itch receptors; instead, they attenuate the pruritogenic background of CKD-aP upstream by improving gut microecology and host inflammatory status.

However, it should be emphasized that current evidence for phytochemicals is mainly derived from mechanistic studies and preclinical data at the level of CKD or the gut–kidney axis (Zheng et al., 2022; Mirmohammadali and Rosenkranz, 2023). At present, there is a lack of well-designed clinical trials evaluating phytochemicals with pruritus improvement as a predefined endpoint in CKD-aP patients. Therefore, phytochemicals should not be described as established antipruritic agents. Instead, they may be considered exploratory adjunctive candidates that could potentially modulate the metabolic and inflammatory background associated with CKD. Their relevance to CKD-aP remains hypothesis-generating and requires validation in prospective human studies. In addition, safety considerations are particularly important in CKD populations. The effective dose, bioavailability, renal handling, herb–drug interactions, product quality, and potential electrolyte or nephrotoxic risks of phytochemical preparations need to be carefully evaluated before clinical application in CKD-aP.

### 5.7 Hormone-like substances

Hormone-like substances, particularly vitamin D and its analogues, have been discussed in relation to CKD-aP (Mousa et al., 2018). Vitamin D signaling is involved in epidermal differentiation, antimicrobial peptide expression, immune regulation, oxidative stress, and neuroimmune communication. Basic and dermatological studies indicate that vitamin D and the vitamin D receptor (VDR) participate in keratinocyte differentiation and skin barrier homeostasis, providing a biological rationale for considering vitamin D-related pathways in chronic pruritic conditions (Bikle, 2012). However, these mechanisms are largely derived from experimental dermatology and broader inflammatory skin disease contexts rather than CKD-aP-specific studies. A small open-label pilot study reported that topical calcipotriol may be a safe and potentially effective candidate for CKD-aP, but its design lacked placebo control and the sample size was limited (Jung et al., 2015). In contrast, a double-blind, placebo-controlled randomized trial evaluating oral ergocalciferol for uremic pruritus did not demonstrate significant efficacy, indicating that systemic vitamin D supplementation should not be assumed to be an effective antipruritic therapy in CKD-aP (Shirazian et al., 2013). More recently, NB-UVB phototherapy has been reported to improve CKD-aP, and the response appeared to correlate with increases in serum vitamin D levels; however, this association does not prove that vitamin D mediates the antipruritic effect of phototherapy (Kee et al., 2024).

Evidence from broader chronic pruritus literature also suggests a possible relationship between vitamin D status or supplementation and pruritus severity, but these analyses include heterogeneous diseases and should be considered supportive background rather than direct CKD-aP evidence (Li et al., 2024). Therefore, vitamin D-related agents should be described as complementary or exploratory approaches that may modify host barrier and immune function, rather than as established gut-directed or microbiome-based therapies for CKD-aP. Their relationship with gut microbial dysbiosis remains indirect and unproven.

Safety considerations are particularly important in CKD and ESKD patients. Vitamin D and vitamin D analogues should be used in the context of CKD-mineral and bone disorder management, with monitoring of serum calcium, phosphate, parathyroid hormone, 25(OH)D levels, and vascular calcification risk. Larger, placebo-controlled trials with standardized dosing, predefined pruritus endpoints, and careful safety monitoring are required to clarify whether vitamin D-related interventions have a clinically meaningful role in CKD-aP management.

## 6 Conclusions and perspectives

CKD-aP is a common and clinically meaningful symptom in patients with CKD and ESKD, with substantial effects on quality of life, sleep, psychological well-being, and clinical outcomes. Its pathophysiology is multifactorial and involves chronic inflammation, immune dysregulation, neural and opioid pathway abnormalities, mineral metabolism disorders, and skin barrier dysfunction. The kidney-gut-skin axis provides a useful conceptual framework for understanding how gut microbial alterations, gut-derived uremic toxins, intestinal barrier impairment, and systemic inflammation may be associated with CKD-aP. However, current evidence is still largely associative or derived from general CKD/ESKD cohorts, animal models, or *in vitro* studies. Therefore, gut microbial dysbiosis should be interpreted as a potential contributor to the pruritogenic milieu rather than as an established causal driver of CKD-aP.

From a clinical perspective, current management should continue to prioritize established approaches, including optimization of dialysis adequacy, correction of mineral metabolism disorders, skin hydration and barrier care, individualized nutritional guidance, and the use of approved or guideline-supported antipruritic therapies when appropriate. Gut-directed or metabolism-directed strategies, including probiotics, prebiotics, synbiotics, AST-120, FMT, phytochemicals, Uremia Clearance Granules, and hormone-related approaches, differ substantially in their evidence levels. Among them, AST-120 and some clinical studies of Uremia Clearance Granules have more direct CKD-aP-related evidence, whereas probiotics, prebiotics, and synbiotics are mainly supported by CKD or dialysis studies in which pruritus was not usually the primary endpoint. FMT, phytochemicals, and many hormone-related interventions remain largely preclinical, indirect, or exploratory. These approaches should therefore be viewed as potential adjunctive strategies rather than established CKD-aP treatments.

Future studies should distinguish CKD-aP-specific findings from general CKD or ESKD-related alterations, control for major microbiome-modifying confounders such as diet, constipation, antibiotics, proton pump inhibitors, phosphate binders, diabetes, and dialysis modality, and incorporate longitudinal designs, multi-omics integration, mechanistic validation, and well-powered randomized controlled trials with pruritus as a predefined endpoint. Such work will help clarify whether specific microbial taxa, metabolites, or gut-derived inflammatory signals play causal roles in CKD-aP and may support the development of more precise and personalized therapeutic strategies.

## Glossary

AGEs: advanced glycation end products
AhR: aryl hydrocarbon receptor
BCP: basic calcium phosphate
BSEP: bile salt export pump
CKD: chronic kidney disease
CKD-aP: chronic kidney disease-associated pruritus
Cr: creatinine
CX3CL1: CX3C chemokine ligand 1
CX3CR1: CX3C chemokine receptor 1
ESKD: end-stage kidney disease
FMT: fecal microbiota transplantation
HD: hemodialysis
MHD: maintenance hemodialysis
GATA3: GATA-binding protein 3
IL-1β: interleukin-1β
IL-2: interleukin-2
IL-6: interleukin-6
IL-12: interleukin-12
IL-31: interleukin-31
iNOS: inducible nitric oxide synthase
IS: indoxyl sulfate
JAK2/STAT3: Janus kinase 2/signal transducer and activator of transcription 3
KOR: κ-opioid receptor
LBP: lipopolysaccharide-binding protein
LPS: lipopolysaccharide
MAMPs: microbial-associated molecular patterns
MOR: μ-opioid receptor
m⁶A: N⁶-methyladenosine
NB-UVB: narrow-band ultraviolet B
NGF: nerve growth factor
NF-κB: nuclear factor-κB
PAR-2: protease-activated receptor 2
PBUTs: protein-bound uremic toxins
pCS: p-cresyl sulfate
p-ERK: phosphorylated extracellular signal-regulated kinase
PD: peritoneal dialysis
PLC-IP3-Ca2+: phospholipase C-inositol trisphosphate-Ca2+
PTH: parathyroid hormone
RCT: randomized controlled trial
ROS: reactive oxygen species
RXR: retinoid X receptorSCFAs, short-chain fatty acids
SynCKD: CKD-specific polybiotic
STAT6: transcription 6
TLR4-MD2: Toll-like receptor 4-myeloid differentiation factor 2
TMAO: trimethylamine N-oxide
TNF-α: tumor necrosis factor-α
TRPV1: transient receptor potential vanilloid 1
TEWL: transepidermal water loss
TGF-β: transforming growth factor-β
Th1: T helper 1
Th2: T helper 2
Th17: T helper 17
Tregs: regulatory T cells
TrkB: tropomyosin receptor kinase B
UCG: Uremia Clearance Granules
VAS: visual analog scale
VDR: vitamin D receptor
ZO-1: zonula occludens-1
